# The complete chloroplast genome of *Castanea sativa* Mill. (Fagaceae)

**DOI:** 10.1080/23802359.2021.1903359

**Published:** 2021-03-28

**Authors:** Cancan Zhu, Shijie Zhang, Xiaoqian Bai, Fuqin Guan, Yu Chen, Yuqiang Zhao, Mimi Li

**Affiliations:** Institute of Botany, Jiangsu Province and Chinese Academy of Sciences, Nanjing, PR China

**Keywords:** *Castanea sativa*, complete chloroplast genome, phylogenetic analysis

## Abstract

*Castanea sativa* Mill. is mainly grown in the temperate regions of continental Europe, and it has a considerable economic value. In this study, the complete chloroplast genome sequence of *Castanea sativa* was characterized. Leaves were collected from the National Botanical Garden of Latvia. The chloroplast genome was determined to be 160,938 bp in length. It contained large single-copy (LSC) and small single-copy (SSC) regions of 90,519 and 18,967 bp, respectively, which were separated by a pair of 25,726 bp inverted repeat (IR) regions. The genome is predicted to contain 130 genes, including 83 protein-coding genes, 37 *tRNA* genes, eight *rRNA* genes, and two pseudo genes. The overall GC content of the genome is 36.8%. A phylogenetic tree reconstructed by 34 chloroplast genomes reveals that *C. sativa* is most closely related to the clade including *C. henryi*, *C. seguinii* and *C. mollissima*.

The genus *Castanea* (Fagaceae) is widely distributed in the deciduous forests of the Northern Hemisphere (Lang et al. 2007). *Castanea sativa* Mill. (Sweet Chestnut) is a broadleaved species with a long-range scattered distribution across the Mediterranean region of Europe and Western Asia. It is an important multipurpose species for nut and timber production. *C. sativa* evolved on the European continent, while species composition and life form diversity of the plant communities have changed markedly both in the abandoned groves and in the periodically clear-cut coppice stands. Over the past few decades, a number of cultivars with good yield and quality were selected (Bostan et al. 2018). In this study, the complete chloroplast genome (cp) of *C. sativa* based on Illumina pair-end sequencing data was characterized for species identification and phylogenetic analysis.

A plant material of *C. sativa* was collected from the National Botanic Garden of Latvia in Salaspils, Latvia (56°51′46.3′′N, 24°21′23.0′′E) in Aug. 2019. Voucher specimen was stored at the Herbarium of the Chestnut Germplasm Resources Repositories in Jiangsu Province, China under No. 190830. The total DNA was extracted from fresh leaves using the DNeasy Plant Mini Kit (Qiagen, Valencia, CA). The whole-genome sequencing was conducted with 150 bp pair-end reads on the Illumina Hiseq X-ten platform (Illumina, San Diego, CA) by Novogene, Beijing, China. With Illumina data, the genome was assembled using NOVOPlasty version 2.7.2 software (Dierckxsens et al. 2017). The annotations of the complete chloroplast genomes were performed with GeSeq (Tillich et al. 2017) and adjusted by manual in Geneious version 11.1.5 (https://www.geneious.com/).

The plastome of *C. sativa* was determined to comprise a double-stranded, circular DNA of 160,938 bp (NCBI acc. no. MW327507), and it contained two inverted repeat (IR) regions of 25,726 bp each, separated by large single-copy (LSC) and small single-copy (SSC) regions of 90,519 and 18,967 bp, respectively. The genome was predicted to contain 83 protein-coding genes, 37 *tRNA* genes, eight *rRNA* genes, and two pseudo genes. The two pseudo genes were *ycf1* gene and *rpl22* gene. Six protein-coding genes, seven *tRNA* genes and four *rRNA* genes were duplicated in IR regions. There were 21 genes containing one intron and two genes (*clp*P and *ycf*3) containing two introns. The overall GC content of *C. sativa* cp genome is 36.8% and the corresponding values in LSC, SSC, and IR regions are 34.6, 30.8, and 42.7%, respectively.

Multiple sequence alignment software, MAFFT version 7.409 (Katoh and Standley 2013) was used to compare 34 representative species. The phylogenetic trees were generated based on maximum-likelihood (ML) approach in RAxML (Stamatakis 2014). The nucleotide substitution model GTR + G were used in phylogenetic tree analysis. The phylogenetic tree indicated that the monophyly of the genus *Castanea* with 100% bootstrap value and *C. sativa* was closely related to the clade including *C. henryi*, *C. seguinii*, and *C mollissima* (Figure 1).

**Figure 1.**
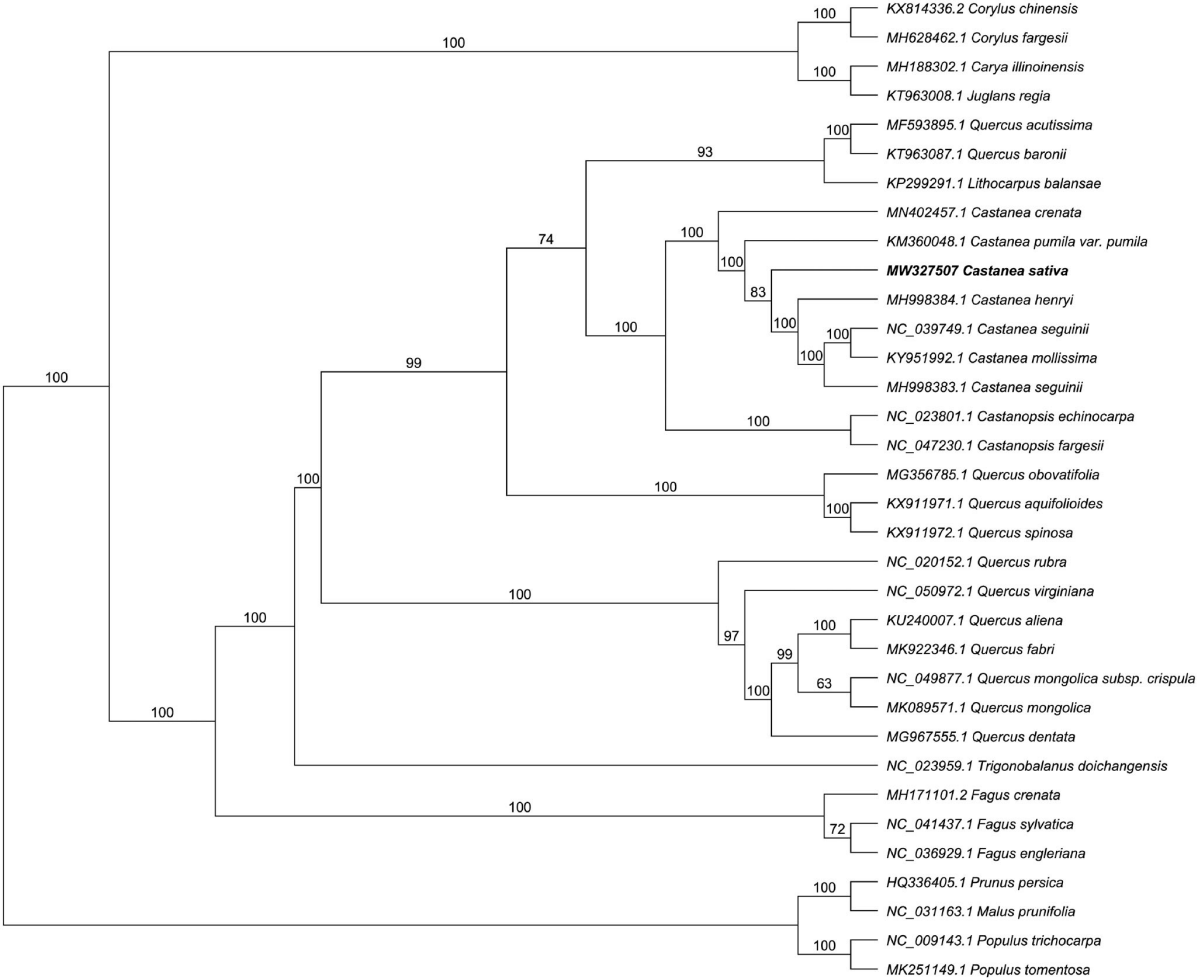
Phylogenetic tree inferred by maximum-likelihood (ML) method based on the complete chloroplast genome of 34 representative species. *Malus prunifolia*, *Populus trichocarpa*, *Populus tomentosa*, and *Prunus persica* were used as the outgroup. A total of 1000 bootstrap replicates were computed and the bootstrap support values are shown at the branches.

## Data Availability

The data that support the findings of this study are openly available in GenBank (https://www.ncbi.nlm.nih.gov) with the accession number is MW327507.
